# Astragalus-containing Chinese herbal medicine used with Western medicine for lupus nephritis: a systematic review and meta-analysis of randomized controlled trials

**DOI:** 10.3389/fphar.2024.1395844

**Published:** 2025-02-21

**Authors:** Jinjiao Li, Mengyun Wu, Weiwei Liu

**Affiliations:** ^1^ Northern Jiangsu people’s Hospital, Yangzhou University, Yangzhou, China; ^2^ The Second Affiliated Hospital of Shandong First Medical University, Taian, Shandong, China

**Keywords:** astragalus, Chinese herbal medicine, lupus nephritis, meta-analysis, efficacy

## Abstract

**Background:**

Lupus nephritis (LN) is a serious complication of systemic lupus erythematosus (SLE) that requires effective management to prevent kidney damage and other systemic effects. While Western medicine provides the standard treatment, incorporating traditional Chinese medicine, such as Astragalus-containing Chinese herbal medicine (CHM), may offer additional benefits in improving patient outcomes.

**Objective:**

This study aims to conduct a systematic review and meta-analysis of the efficacy and safety of Astragalus in conjunction with Western medicine for the treatment of LN.

**Methods:**

We conducted a comprehensive, global systematic search across databases including PubMed, Web of Science, Embase, China National Knowledge Infrastructure (CNKI), and Wanfang. Data were synthesized using fixed- or random-effects models, depending on the level of heterogeneity. Results were presented as standardized mean difference (SMD), risk ratios (RRs), or number needed to treat (NNT) with 95% confidence intervals (CIs). The Cochrane Q test and I^2^ statistics were used to test the heterogeneity assessment. Trial sequential analysis (TSA) was employed to assess the power of the results. All statistical analysis was carried out using STATA (version 16.0).

**Result:**

The analysis included 14 RCTs, with a total of 800 patients (417 in the treatment group and 383 in the control group). Our meta-analysis revealed that patients treated with Astragalus-containing CHM alongside Western medicine showed markedly improved outcomes compared to those receiving only Western medicine. Significant improvements were observed in Systemic Lupus Erythematosus Disease Activity Index (SLEDAI) scores (SMD = 1.01, 95% CI: 0.71–1.30, P < 0.001), 24-h proteinuria (SMD = 0.51, 95% CI: 0.35–0.66, P < 0.001SMD = 0.51, 95% CI: 0.35–0.66, P < 0.001), serum creatinine (SCr) levels (SMD = 0.64, 95%CI: 0.27–1.01, P < 0.001), blood urea nitrogen (BUN) levels (SMD = 0.73, 95%CI: 0.53–0.92, P < 0.001), and overall response rates (ORR) (RR = 1.21, 95%CI: 1.10–1.34, P < 0.001). Additionally, the incidence of adverse events (AEs), such as diarrhea, fever, and other symptoms, was significantly lower in the group treated with Astragalus-containing CHM and Western medicine (RR = 0.56, 95%CI: 0.42–0.73, P < 0.001). The TSA indicated that the data were sufficiently robust to draw reliable numerical conclusions regarding the ORR and the incidence of AEs.

**Conclusion:**

The inclusion of Astragalus-containing CHM alongside Western medicine may be a promising strategy for to improve the therapeutic effectiveness and reduce toxicity in the treatment of LN.

## Introduction

Systemic lupus erythematosus (SLE) is a chronic autoimmune disorder characterized by aberrant lymphocyte activation and subsequent overproduction of autoantibodies. These autoantibodies can adversely affect multiple organ systems (Kuhn et al., 2015). In China, lupus nephritis (LN), a serious sequela of glomerular disease, affects more than half of the adult SLE population (Zhang et al., 2021). Given the significant burden of LN, novel and effective therapeutic strategies that regulate immune function and control inflammatory responses are of paramount importance.

Traditional Chinese Herbal Medicine (CHM) has been increasingly recognized for its potential therapeutic benefits in LN management (Liu et al., 2022; Wang et al., 2022). Clinical trials have progressively supported the efficacy of CHM in LN treatment (Liu et al., 2022). Astragalus, also known as ‘Astragali Radix’ or ‘Huangqi,’ derived from the dried roots of Astragalus membranaceus variants, is noteworthy in this context (Zhang et al., 2014), has been used to treat LN. Rooted in CHM principles, Astragalus is believed to improve Qi, enhance bodily functions, strengthen the body’s defenses, reduce perspiration, and promote fluid secretion (Agyemang et al., 2013; Zheng et al., 2020). Additionally, it has demonstrated potential to reduce hematuria and proteinuria, lower blood pressure, alleviate edema, and provide renal protection (Zhou et al., 2020; Shi et al., 2024). Its application extends to various conditions like constipation, lung disease, blood clots, white turbidity, edema, fetal restlessness, alcohol poisoning, and glomerulonephritis (Zhou et al., 2018; Chen et al., 2020; Sun et al., 2020; Yang et al., 2023).

In CHM, the recommended dosage of Astragalus (Huangqi) can vary significantly based on the condition being treated and the individual patient’s characteristics. For conditions such as general immune enhancement or fatigue related to Qi deficiency, the dosage typically ranges from 9 to 30 g per day. However, in more severe conditions like nephrotic syndromes or LN, the dosage may be increased to 30–60 g per day, particularly when used as part of a decoction or in combination with other herbs aimed at restoring kidney function and alleviating proteinuria (Shi et al., 2024). In clinical practice, the exact dosage is often tailored to the patient’s specific syndrome, as guided by CHM principles, which emphasize individualization of treatment based on the patient’s overall constitution and presenting symptoms.

Recent studies have increasingly highlighted the efficacy of Astragalus-containing CHMs as adjunctive treatments in LN (Su et al., 2007; Chen et al., 2022; Kong et al., 2024). However, the results of different studies have been inconsistent. This study aims to systematically evaluate the potential benefits and adverse effects of Astragalus-containing CHM in conjunction with Western medicine in the treatment of LN.

## Methods

This study was performed in according to the guidance of the Preferred Reporting Items for Systematic Reviews and Meta-Analyses (PRISMA) statement (Moher et al., 2009) and the PRISMA checklist is presented in Supplementary Material 1.

### Literature search strategy

We conducted comprehensive searches in several electronic databases, including PubMed, Embase, Web Of Science, China National Knowledge Infrastructure (CNKI) and Wanfang from their inception to 14 February 2024. In addition, we performed manual searches by examining all references, exploring grey literature, and reviewing theses, government documents, letters, abstracts, minutes of meetings, and research reports to mitigate potential publication bias. Our search strategy primarily used keywords such as “Lupus nephritis,” “Huangqi,” “Astragalus,” and “Chinese herbal medicine” to identify relevant studies (Supplementary Material 2).

### Inclusion criteria

Studies were eligibility in our meta-analysis if they met the following criteria: (1) Patients: Participants must meet the classification criteria for LN as outlined by the American College of Rheumatology (ACR) in 1997. (2) Intervention: Participants in the treatment group must have received treatment involving CHM containing Astragalus, administered in various forms including capsules, tablets, decoctions, or intravenous administration, in combination with Western medicine. (3) Control: The control group must have been administered with Western medicine only. (4) Outcome Measures: The selected studies must have reported relevant outcome measures, including SLEDAI score, 24-h urinary protein quantification (24h-PRO), serum creatinine (SCr) levels, overall response rate (ORR), blood urea nitrogen (BUN) levels, and adverse events (AEs). (5) Study design: Only RCTs were eligible for inclusion.

### Data extraction

Two independent investigators extracted the following data and information: (1) Identification Information: This included the year of publication and the first author’s name (2) General Information: Pertaining to the study, including the setting, sample size, and period of follow-up. (3) Participant Details: Information such as the age and sex of the participants. (4) Intervention Specifics: Details about the CHM intervention, including its name, composition, and duration of administration. (5) Comparison Details: Information about the Western medicine regimen used in the control group, including the dose, frequency, and duration of treatment. (6) Outcome Details: Data on the various outcomes measured in the study. In cases where there were disagreements between our two primary reviewers, we attempted to resolve them through dialog. If an agreement could not be reached, we included in a third reviewer to facilitate a consensus.

### Quality assessment

The risk of bias in the included studies was assessed using the Cochrane Risk of Bias Tool for RCTs (RoB 2) (Sterne et al., 2019). The RoB 2 tool evaluates the methodological quality of randomized trials across five domains: (1) bias arising from the randomization process, (2) bias due to deviations from intended interventions, (3) bias due to missing outcome data, (4) bias in measurement of the outcome, and (5) bias in selection of the reported result. Each domain was rated as having a low, high, or unclear risk of bias based on the available information.

### Statistical analysis

We calculated the risk ratio (RR) and number needed to treat (NNT) with 95% confidence interval (CI) for dichotomous data. For continuous variables, we used the standardized mean difference (SMD) with a 95%CI. The primary outcome was the SLEDAI score, a numerical variable reflecting disease activity. The secondary outcomes included the 24h-PRO (numerical variable), SCr levels (numerical variable), BUN levels (numerical variable), ORR (categorical variable) and AE (categorical variable). SMD was used for the synthesis of continuous data because the included studies reported outcomes using varying measurement units. In cases where all studies for a specific outcome used consistent units, the mean difference (MD) was considered the preferred approach for data synthesis. However, such consistency was not observed for all the continuous outcomes, necessitating the use of SMD to standardize the results. To assess heterogeneity among the included studies, we employed the Cochrane Q statistic and *I* ^2^ statistic (Higgins et al., 2003). Heterogeneity was considered significant if the p-value was less than 0.1 or *I* ^2^ exceeded 50% (Higgins et al., 2003). Considering that the inclusion of multiple polyherbal formulas containing Astragali Radix introduces inherent heterogeneity into the interventions, only a random-effects models is employed for the analysis across all the outcomes. The Begg (Begg and Mazumdar, 1994) and Egger’s (Egger et al., 1997) tests were used to test the publication bias. A P value <0.05 was considered statistically significant unless a specific p-value threshold had been specified. STATA version 16.0 executed all statistical analyses (Stata Corporation, College Station, TX, United States).

### Meta-regression analyses

In order to account for heterogeneity observed across the included studies, we hypothesized that variations might be associated with the sample size and treatment duration. To explore the potential influence of these factors on the observed outcomes, we conducted meta-regression analyses. In these analyses, the outcomes were set as dependent variables (y), while the aforementioned covariates (sample size and treatment duration) were treated as independent variables (χ). For analytical purposes, we categorized these variables into groups: sample size as less than 50 or 50 and above, and treatment duration as less than 3 months or 3 months and above.

### Trial sequential analysis

Trial Sequential Analysis (TSA) was used as a methodological tool to refine the statistical significance thresholds in our meta-analysis. This adjustment is crucial in mitigating the risk of random errors that may arise from sparse data and repetitive testing. TSA functions similarly to calculating a sample size in a single study by determining the required information size (RIS) to enhance the accuracy of statistical conclusions drawn from a meta-analysis. For this analysis, we used TSA software, setting a 5% risk for type I error (α = 0.05) and a power of 80% (β = 0.20, indicating a 20% risk of type II error). The expected effect of the intervention was derived from the effect sizes observed in the studies included in our review.

## Results

### Study selection

**FIGURE 1:**
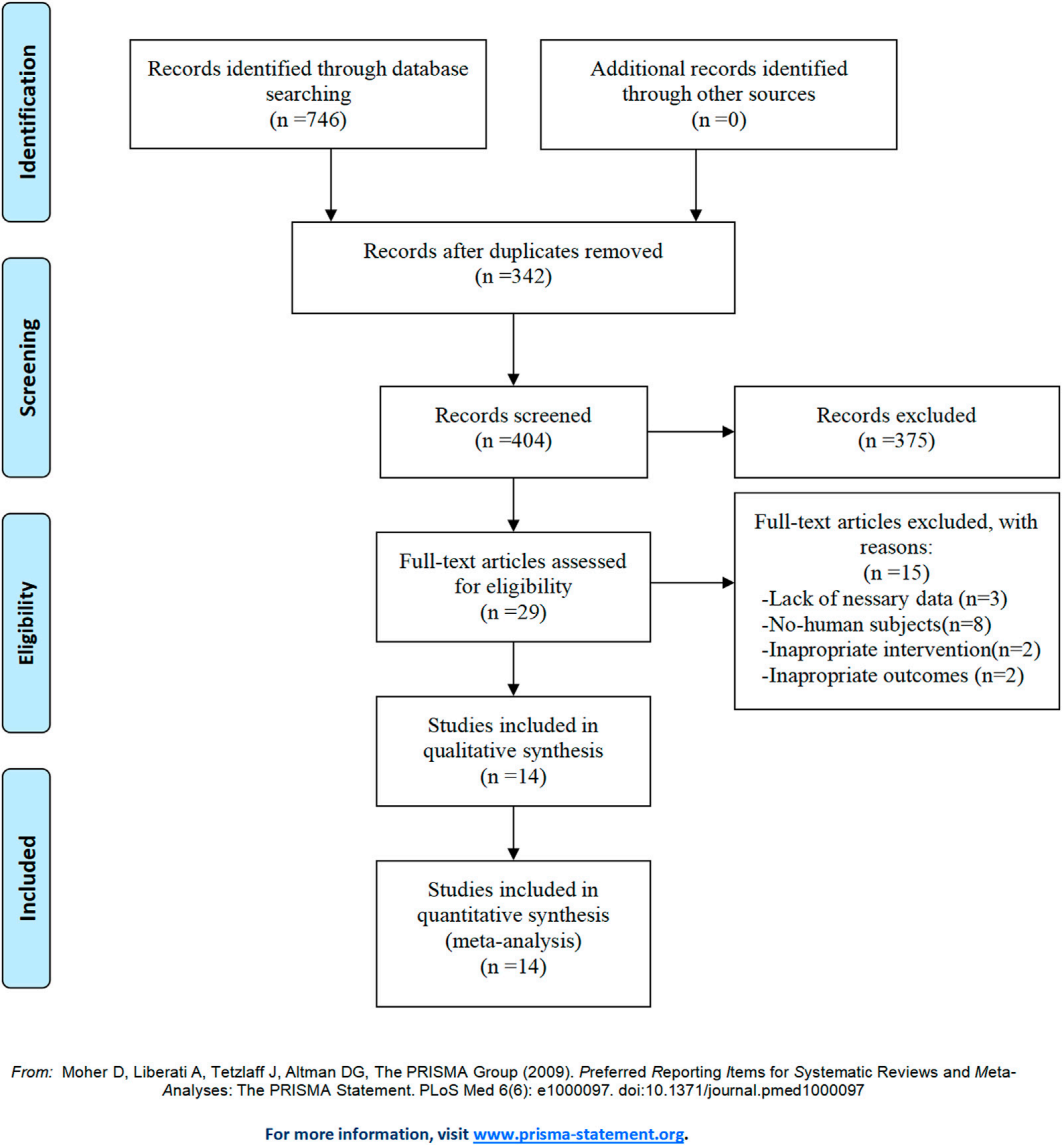
PRISMA flowchart illustrating the study selection and screening process. The initial search yielded 746 records. After removing duplicates, 404 studies were assessed for title and abstract, of which 375 were excluded based on relevance and eligibility criteria. The remaining 29 studies were assessed for full text, resulting in 15 studies being excluded due to reasons such as lack of necessary data, non-human subjects, and inappropriate interventions or outcomes. Ultimately, 14 studies were included in the meta-analysis.

Figure 1 shows the search strategy and selection process for the meta-analysis. Initially, our search yielded a total of 746 studies. After removing 342 duplicate records, 404 studies remained for title and abstract review. Of these, 375 were excluded for various reasons. Subsequently, the remaining 29 studies were then screened for full-text. However, 15 studies were excluded due to various reasons, including lack of necessary data (n = 3), non-human subjects (n = 8), and inappropriate interventions (n = 2) or outcomes (n = 2). Ultimately, 14 studies (Chen et al., 2003; Li et al., 2007; Su et al., 2007; Zhong et al., 2007; Xiang et al., 2008; Zhang, 2008; Qu et al., 2010; Wang et al., 2010; Xie, 2010; Lu, 2019; Yang and Su, 2019; Yuan et al., 2021; Ge et al., 2022; Gui, 2022) were confirmed to meet the inclusion criteria. Therefore, they were therefore included in the meta-analysis.

### Study characteristics of included studies

Table 1 shows summaries of the main characteristics of the studies included in the meta-analyses. All the studies were conducted in China, spanning from 2003 to 2023. Our analysis encompassed a total of 800 patients from 14 studies, with 417 in the group receiving Astragalus-containing CHM in combination with Western medicine, and 383 in the Western medicine group. All 14 studies included in the meta-analysis employed a control group that received standard Western medicine treatment for LN. Each study included a distinct control group, and no studies shared control groups. The intervention regimens varied significantly and included combinations such as Astragalus-containing CHM + GC (Glucocorticoid) + MMF (Mycophenolate Mofetil), Astragalus-containing CHM + GC + CTX (Cyclophosphamide), Astragalus-containing CHM + GC, Astragalus-containing CHM + GC + LEF (Leflunomide), and Astragalus-containing CHM + GC + HCQ (Hydroxychloroquine). In contrast, the control regimens encompassed treatments like GC + MMF, GC + CTX, GC, MMF, GC + LEF, and GC + HCQ. Treatment duration ranged from 3 months to 6 months. Moreover, a detailed table summarizing the herbal medicine regimens used in the intervention group in each RCT is provided in Supplementary Material 3.

**TABLE 1:** Characteristics of included studies.

Study	Sample size	Intervention group	Control group	Outcomes	Duration
Guishiyuan 2022	31/31	Astragalus-containing CHM+GC+MMF	GC+MMF	①②③④⑤	3 months
Zhu Aimin 2010	32/30	Astragalus-containing CHM+GC+CTX	GC+CTX	②③	—
Qu Huanru 2010	15/15	Astragalus-containing CHM+GC+CTX	GC+CTX	①②⑤	3 months
Xie Chao 2010	23/23	Astragalus-containing CHM+GC+CTX	GC+CTX	①②③④	2 months
Li Gui’an 2006	50/41	Astragalus-containing CHM+GC+CTX	GC+CTX	④⑤	3 months
Zhang Minghua 2008	20/18	Astragalus-containing CHM+GC	GC	④⑤	—
Geyang 2022	30/30	Astragalus-containing CHM+MMF	MMF	①②③④	3 months
Xiang Caichun 2008	30/30	Astragalus-containing CHM+GC+CTX	GC+CTX	②③④⑤	3 months
Lu Sihao 2019	15/15	Astragalus-containing CHM+GC+LEF	GC+LEF	②③④⑤	6 months
Yang Yeying 2019	29/26	Astragalus-containing CHM+GC+HCQ	GC+HCQ	①②③⑤	3 months
Chen Xiangjun 2003	37/22	Astragalus-containing CHM+GC	GC	②⑤	3 months
Su Li 2007	23/20	Astragalus-containing CHM+GC	GC	②	—
Zhong Li 2007	50/50	Astragalus-containing CHM+GC	GC	②③⑤	—
Yuan Xiaoying 2021	32/32	Astragalus-containing CHM+GC+MMF	GC+MMF	①③④⑤	—

Abbreviation: CHM: Chinese herbal medicine; GC: Glucocorticoid; MMF: Mycophenolate Mofetil; CTX: Cyclophosphamide; LEF: Leflunomide; HCQ: Hydroxychloroquine.

### Risk of bias assessment

**FIGURE 2:**
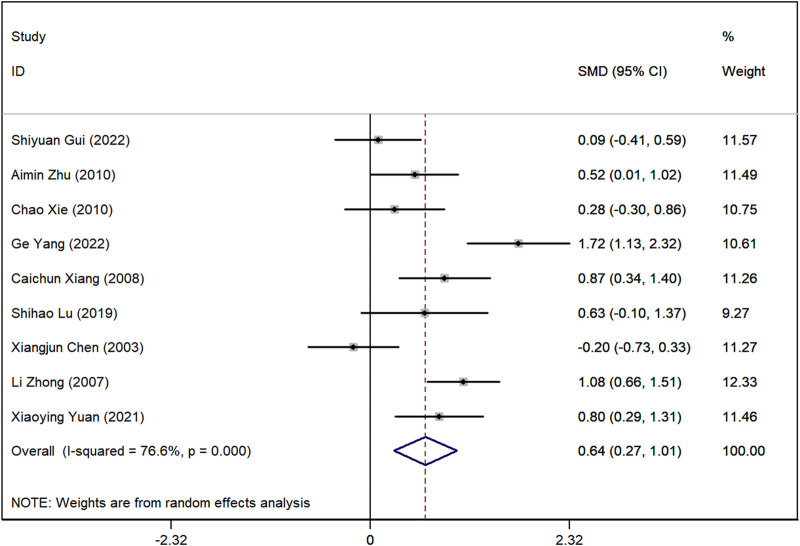
Risk of bias assessment using the Cochrane Risk of Bias tool for the 14 randomized controlled trials (RCTs) included in this meta-analysis. The figure categorizes each study based on the risk of bias (low, unclear, or high) in various domains: random sequence generation, allocation concealment, blinding of participants and personnel, blinding of outcome assessment, incomplete outcome data, and selective reporting. A total of one study was considered to have a low risk of bias, 11 studies had an unclear risk, and two studies had a high risk of bias.

The methodological quality of fourteen RCTs was assessed using the ROB2 (Figure 2). Of the 14 studies, one study (Lu, 2019) was assessed as having a low risk of bias across all the domains. Eleven studies (Chen et al., 2003; Li et al., 2007; Su et al., 2007; Zhong et al., 2007; Xiang et al., 2008; Wang et al., 2010; Xie, 2010; Yang and Su, 2019; Yuan et al., 2021; Ge et al., 2022; Gui, 2022) were categorized as having “some concerns” regarding the risk of bias, and two studies (Zhang, 2008; Qu et al., 2010) was deemed to have a high risk of bias due to the absence of allocation concealment and blinding in the randomization process.

### SLEDAI score

**FIGURE 3:**
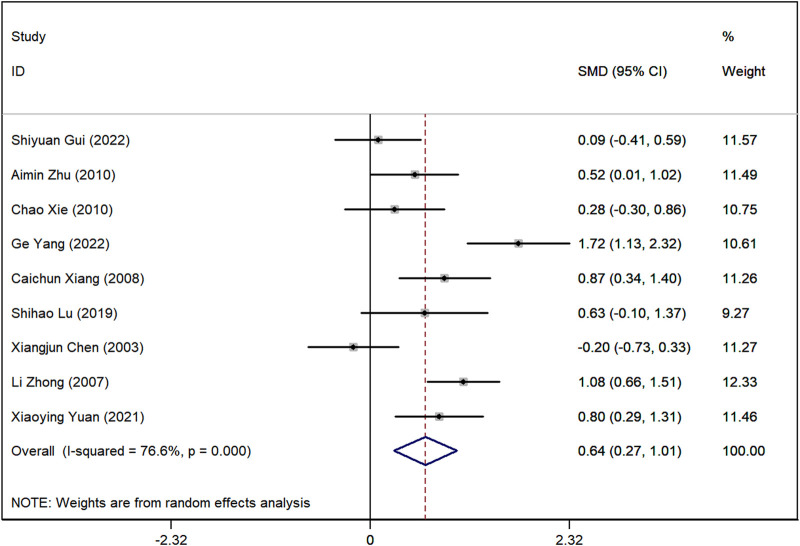
Forest plot showing the standardized mean difference (SMD) in the Systemic Lupus Erythematosus Disease Activity Index (SLEDAI) between patients treated with Astragalus-containing Chinese herbal medicine (CHM) in combination with Western medicine versus those treated with Western medicine alone. A significant reduction in the SLEDAI score was observed in the combined treatment group (SMD = 0.68, 95% CI: 0.38–0.98, P < 0.001). The plot also presents the individual study results and the overall pooled estimate, with moderate heterogeneity (I^2^ = 41.2%).

Data on SLEDAI scores were available from six studies. The meta-analysis revealed a significant difference, demonstrating that the SLEDAI score was significantly lower in the group receiving Astragalus-containing CHM in conjunction with Western medicine compared with the group receiving Western medicine alone (SMD = 1.01, 95% CI: 0.71–1.30, P < 0.001). The heterogeneity across these studies was moderate (I^2^ = 33.3%) (Figure 3).

### 24-h urinary protein quantification (24h-PRO)

**FIGURE 4:**
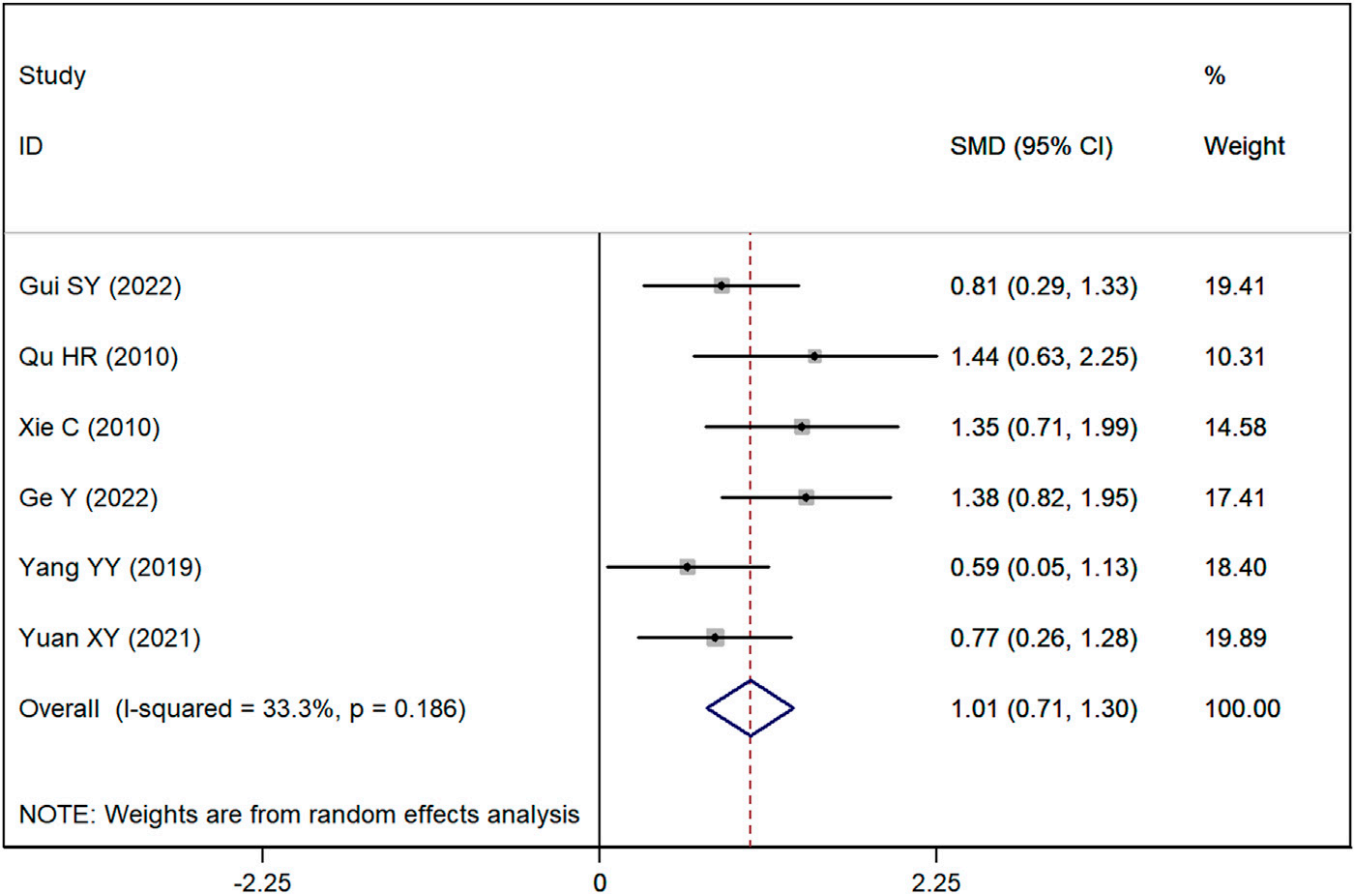
Forest plot demonstrating the standardized mean difference (SMD) in 24-h urinary protein (24h-PRO) levels between patients treated with Astragalus-containing Chinese herbal medicine (CHM) combined with Western treatment and those treated with Western treatment alone. A significant reduction in proteinuria was found in the combined treatment group (SMD = 0.51, 95% CI: 0.35–0.66, P < 0.001). The heterogeneity across studies was low (I^2^ = 0.0%).

Twelve studies reported data on 24-h proteinuria (24h PRO). The meta-analysis revealed a notable difference, with the 24h PRO levels being significantly lower in the group treated with Astragalus-containing CHM plus Western medicine than in the Western medicine group (SMD = 0.51, 95%CI: 0.35–0.66, P < 0.001). The test for heterogeneity was not significant (I^2^ = 0.0%) (Figure 4).

### Serum creatinine (SCr) levels

**FIGURE 5:**
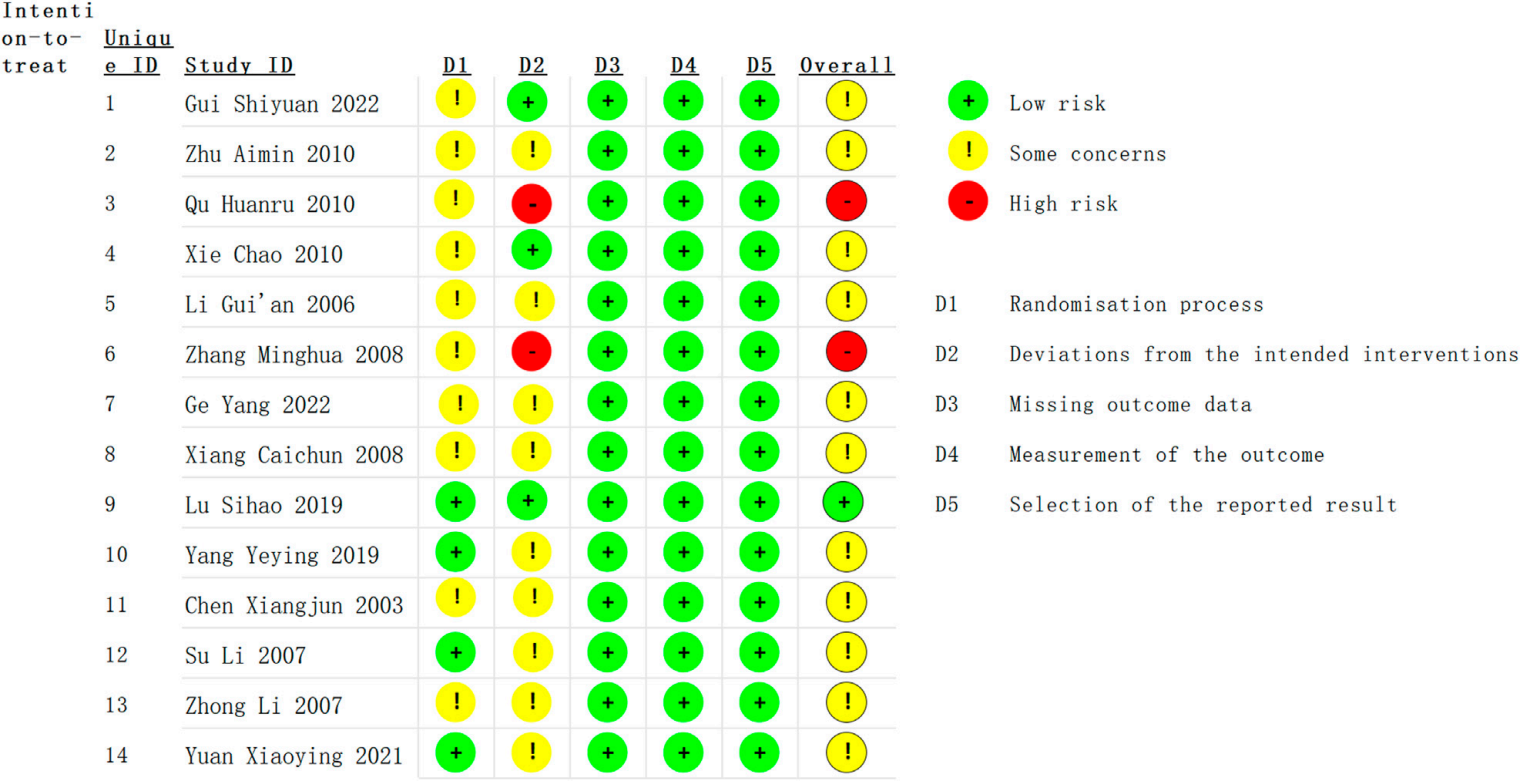
Forest plot illustrating the standardized mean difference (SMD) in serum creatinine (SCr) levels between patients receiving Astragalus-containing Chinese herbal medicine (CHM) combined with Western medicine and those receiving Western medicine alone. The combined treatment group showed significantly lower serum creatinine levels (SMD = 0.62, 95% CI: 0.26–0.97, P < 0.001). However, substantial heterogeneity was observed (I^2^ = 74.7%).

Data on SCr levels were reported in nine studies. The analysis showed a marked difference, with lower SCr levels in the group receiving Astragalus-containing CHM alongside Western medicine compared to the Western-only group (SMD = 0.64, 95%CI: 0.27–1.01, P < 0.001) (Figure 5). However, significant heterogeneity was observed among these studies (I^2^ = 76.7%). Sensitivity analyses indicated that, regardless of the exclusion of any individual study, the lower and upper limits of the 95% CI for the pooled effect size of the remaining studies were 0.18 and 1.10, respectively (Supplementary Material 4). As neither limit crossed 0, these results suggest that the exclusion of any single article would not alter the overall effect estimate, confirming the robustness of the meta-analysis findings.

### Blood urea nitrogen (BUN) levels

Seven studies reported BUN levels. The meta-analysis using a random-effects model showed that BUN levels were significantly lower in the group treated with Astragalus-containing CHM and Western medicine than in the group treated with Western treatment alone (SMD = 0.73, 95%CI: 0.53–0.92, P < 0.001) (Supplementary Material 5). There was no significant heterogeneity among the studies (I^2^ = 0.0%).

### Overall response rate (ORR)

ORR data were available from eight studies. The meta-analysis showed that the ORR was significantly higher in the group receiving Astragalus-containing CHM with Western medicine compared with the Western medicine group (RR = 1.21, 95%CI: 1.10–1.34, P < 0.001) (Supplementary Material 6). This indicates that patients treated with the combined therapy were 21% more likely to achieve a positive treatment response compared to those in the Western medicine control group. Moderate heterogeneity was observed (I^2^ = 38.0%). The NNT for the ORR between the intervention group and the control group was 5.56 (95% CI: 3.87–13.17). This indicates that, on average, treating approximately six patients with the combined therapy would result in one additional patient achieving a better treatment response compared to using Western medicine alone.

### Adverse events (AEs)

Ten studies reported data on AEs. The incidence of AEs was significantly lower in the group treated with Astragalus-containing CHM and Western medicine than in the group treated with Western treatment alone (RR = 0.56, 95%CI: 0.42–0.73, P < 0.001) (Supplementary Material 7). The heterogeneity among these studies was not significant (I^2^ = 8.5%).

### Trial sequential analysis (TSA)

The cumulative sample sizes for ORR and AE exceeded the optimum sample size for TSA (Supplementary Material 8). For ORR, the cumulative Z-curve crossed the trial sequential monitoring boundary for benefit, providing firm evidence of a 23% increase in ORR with Astragalus-containing CHM with Western treatment compared with the Western treatment (Supplementary Material 8A). Additionally, the Z-curve surpassed the RIS of 503 patients, suggesting that the analysis was adequately powered to detect a clinically meaningful difference in ORR and that further trials may not be necessary to confirm this benefit. For AE, the cumulative Z-curve crossed the trial sequential monitoring boundary for benefit, substantiating the conclusion that the Astragalus-containing CHM with Western treatment resulted in a substantial decrease in incidence of AEs compared with the Western treatment. In addition, the z-curve exceeded the RIS of 643 participants (Supplementary Material 8B). This suggests that the present study incorporated a sufficient number of patients to reach a statistically reliable conclusion and that additional studies may not be necessary to confirm this benefit.

### Meta-regression analyses

Meta-regression analyses were performed for Scr level and BUN levels. Meta-regression analysis showed no significant correlation between sample size and Scr level (coefficient = 0.22, 95% CI: −1.65–2.09; P = 0.791) or BUN level (coefficient = −0.28, 95% CI: −4.32–3.77; P = 0.868). Similarly, the meta-regression analysis of treatment duration also revealed no significant correlation with Scr level (coefficient = 0.31, 95% CI: −1.21–1.83; P = 0.649) or BUN level (coefficient = −0.41, 95% CI: −3.19–2.38; P = 0.723). This indicated that the sample size of the studies and the duration of treatment did not have a significant effect on the measured Scr level and BUN level.

### Assessment of publication bias

The result of the publication bias assessment showed that there was no significant publication bias among the included studies (Begg’s test: P = 0.244; Egge’s test: P = 0.594).

## Discussion

This meta-analysis rigorously examines the efficacy and safety of Astragalus-containing CHM in the treatment of LN. Our results demonstrate a significant improvement in treatment efficacy with the addition of Astragalus to Western medicine. Significant improvements were observed in several clinical metrics, including the SLEDAI score, 24-h proteinuria, SCr levels, and BUN levels. Additionally, the ORR was significantly higher in the group receiving the combined treatment group. Importantly, this group also exhibited a reduced incidence of AEs compared to the control group receiving only Western medicine.

Previous studies have highlighted Astragalus for its diuretic effects, ability to increase serum protein levels, reduce urinary protein excretion, improve blood cell counts, and modulate the immune system (Liu et al., 2017). Both TCM and Western medical literature provide theoretical and experimental support the use of Astragalus in the treatment of kidney disease. Astragalus components are known to attenuate inflammation through various signaling pathways, alleviate podocyte damage, inhibit renal fibrosis, and thus, slow the progression of kidney damage. Clinical studies highlight the efficacy of Astragalus’s effectiveness in controlling key pathogenic indicators of kidney diseases, such as urinary protein levels, blood urea nitrogen, Scr, and blood uric acid (Li et al., 2017; Zhang J. et al., 2020). This control is crucial in managing and decelerating the progression of kidney diseases.

However, there is a lack of evidence-based medicine for LN. An increasing body of literature suggests that LN leads to substantial protein loss, and many patients exhibit symptoms indicative of qi deficiency, a term in CHM denoting insufficient vital energy and normal immune regulatory capacity (Hong, 2012). These symptoms can potentially be alleviated through the use of Astragalus (Wang et al., 2022). In CHM, this effect is referred to as ‘tonify qi’ (Gao et al., 2018; Deng et al., 2021). Recent studies have also demonstrated that Astragalus regulates the number of Th17 cells and modulates cytokine levels, including tumor necrosis factor α, interleukin (IL)-12, interferon-gamma, and IL-17A in mouse serum, resulting in the inhibition of LN progression (Chen et al., 2022). Astragali Radix suppresses key pro-inflammatory cytokines, including TNF-α, IL-6, IL-17, and IFN-γ(Lee et al., 2024), which are pivotal in SLE pathogenesis. Moreover, Astragali Radix exhibits anti-inflammatory and immunomodulatory effects by modulating the PI3K/AKT/mTOR pathway (Zhan et al., 2024), suggesting its potential as a therapeutic target. Its total flavonoids enhance macrophage activity and regulate cytokine production, contributing to both anti-inflammatory and immunomodulatory responses (Guo et al., 2016). Additionally, Astragali Radix influences critical pathways, such as NF-κB, IL-17, and Toll-like receptors, which are central to inflammatory and immune mechanisms, further underscoring its promise in SLE treatment (Hua et al., 2024). Our study confirmed that Astragalus can effectively lower the SLEDAI score and reduce urinary protein levels in LN patients. These results showed significant statistical significance, highlighting its therapeutic efficacy.

The conventional LN treatment regimen typically involves glucocorticoids combined with immunosuppressants, such as cyclophosphamide, or biological agents such as rituximab and belimumab (Illei et al., 2001; Furie et al., 2022; Parodis and Houssiau, 2022; Dou et al., 2023). In severe cases, treatment may extend to plasma exchange or gamma globulin administration (Kronbichler et al., 2019; Arora and Rovin, 2022). However, these potent medications often carry the risk of side effects affecting multiple various organ systems, including musculoskeletal, gastrointestinal, cardiovascular, endocrine, neuropsychiatric, dermatologic, ocular, and immunologic functions (Oray et al., 2016; Mathian et al., 2023). Notably, LN patients often have increased production of vascular endothelial growth factor (VEGF) production in the kidneys, resulting in increased blood VEGF levels (Avihingsanon et al., 2009; Feliers, 2009). VEGF is predominantly expressed in renal podocytes and collecting ducts, with lower expression in healthy controls (Avihingsanon et al., 2009). This observation might explain the improved renal response in patients treated with Astragalus-containing CHM in conjunction with Western medicine, compared to those treated with Western medicine alone. Crucially, managing renal flares is integral to the prevention of adverse outcomes in LN patients. Our study also found that the incidence of adverse effects was lower in patients receiving the combined treatment of Astragalus-containing CHM and Western medicine.

Significant fatigue is reported by two-thirds of SLE patients and is a common and profoundly limiting symptom. This fatigue is often perceived as a qi deficiency from a Chinese medicine perspective (Bakshi et al., 2018). Cross-sectional studies have shown that muscle weakness correlates with poorer physical function in female SLE patients. In C26 colon cancer cachexia mouse models, a standardized herbal combination of Astragalus and Peony has been shown to have a protective impact against muscle atrophy as reported by certain studies (Lee et al., 2021). Further research is essential to determine whether Astragalus may alleviate muscle weakness in individuals with lupus. Researchers have also uncovered the efficacy and safety of Astragalus in treating Idiopathic Pulmonary Fibrosis (IPF), leading to improvements in lung function and exercise tolerance among IPF patients (Zhang Y. et al., 2020). This accumulating body of evidence suggests that Astragalus may have unknown therapeutic potentials that have yet to be explored.

It is important to emphasize that when treating LN with Astragalus-containing CHM, careful attention should be paid to syndrome differentiation and treatment. This approach has proven effective for patients presenting with Qi deficiency syndrome, characterized by symptoms such as shortness of breath, fatigue, mental fatigue, and a deficient pulse. TCM principles, such as Qi tonification, are central to the theoretical framework behind Astragalus use. Astragalus has been shown to exert anti-inflammatory and immunomodulatory effects through the inhibition of pro-inflammatory cytokines, reduction of oxidative stress, and renal protection (Chen et al., 2022; Wang et al., 2022). These mechanisms may contribute to its observed efficacy in reducing proteinuria and improving renal function in LN patients. It is noteworthy that a significant proportion of SLE patients may manifest symptoms of Qi deficiency syndrome due to the protracted course of their illness and proteinuria loss. However, it is imperative to acknowledge that this method is not universally applicable and does not encompass all patients. For individuals whose clinical presentation does not align with Qi deficiency syndrome, the use of Astragalus should either be avoided or used with caution, subject to the removal of empirical evidence supporting its suitability.

While our study provides valuable insights, it is important to acknowledge several limitations. Firstly, inherent heterogeneity among the included studies in terms of patient characteristics, disease stages, and treatment approaches may have influenced our results. Although we conducted sensitivity and meta-regression analyses to explore this heterogeneity, no significant influencing factors were identified. Further larger-scale, well-designed RCTs are required to confirm these results. A second limitation pertains to the reporting quality within the source studies. Among the 14 studies analyzed, only two described their methods of allocation concealment and blinding. The majority lacked comprehensive reporting on randomization, allocation concealment, and blinding, thereby introducing a potential bias into our analysis. Thirdly, all 14 RCTs included in this meta-analysis were conducted in China, which raises concerns about the generalizability of our findings to broader populations, particularly those outside China. Cultural and dietary differences, along with potential genetic variations in drug metabolism, may affect the treatment outcomes in different ethnic populations. Fourthly, the clinical practice of TCM is often deeply rooted in region-specific diagnostic and therapeutic principles, which may limit the applicability of the results in Western medical settings. Therefore, while the results are promising, caution should be exercised when applying these findings to non-Chinese populations. Furthermore, the protocol for this meta-analysis was not pre-registered in a recognized database such as PROSPERO prior to its commencement. Pre-registration of systematic review and meta-analysis protocols is an important practice to ensure transparency, reduce bias, and promote methodological rigor. While we have provided a detailed description of our methodology, including study selection criteria, data analysis plan, and risk of bias assessment, we acknowledge that future studies would benefit from protocol pre-registration to further enhance the transparency and reproducibility of the research process. Lastly, treatment duration and dosage were not consistent across the included studies, which may have contributed to heterogeneity. Due to the limited data available, we were unable to perform subgroup analyses for treatment duration and dosage.

In conclusion, our systematic review and meta-analysis suggest that the combination of Astragalus-containing CHM with Western medicine may offer a promising therapeutic strategy for improving treatment outcomes and reducing AEs in patients with LN. However, the current evidence is predominantly based on studies conducted in China, which limits the generalizability of these findings to populations outside of China. To validate these results and assess the global applicability of this treatment approach, there is a clear need for larger, well-designed RCTs conducted across diverse geographical and ethnic populations. Such studies will be critical in determining whether Astragalus-containing CHM can be integrated into worldwide clinical practice for the management of LN.

## Data Availability

The raw data supporting the conclusions of this article will be made available by the authors, without undue reservation.

## Abbreviations

ACR, American College of Rheumatology; AEs, Adverse events; BUN, Blood urea nitrogen; CHM, Chinese herbal medicine; CI, Confidence Interval; CNKI, China National Knowledge Infrastructure; CTX, Cyclophosphamide; SLE, Systemic lupus erythematosus; GC, Glucocorticoid; HCQ, Hydroxychloroquine; LEF, Leflunomide; LN, lupus nephritis; MMF, Mycophenolate Mofetil; 24h-PRO, 24-h urinary protein quantification; PRISMA, Preferred reporting items for systematic reviews and meta-analyses; RCTs, randomized controlled trials; RR, Risk Ratio; SCr, Serum creatinine; SLEDAI, Systemic Lupus Erythematosus Disease Activity Index; SMD, Standardized Mean Difference; TCHM, Traditional Chinese herbal medicine.
