# Supplementary material for: Astragalus-containing Chinese herbal medicine used with Western medicine for lupus nephritis: a systematic review and meta-analysis of randomized controlled trials
Source: Front Pharmacol. 2025 Feb 21;15:1395844. doi: 10.3389/fphar.2024.1395844 (PMC11885490; doi:10.3389/fphar.2024.1395844)
Supplement: Supplementary file 1 [file DataSheet1.docx]

**Supplementary file 1: PRISMA 2020 checklist**

| Section and Topic | Item # | Checklist item | Location where item is reported |
| --- | --- | --- | --- |
| TITLE | | |  |
| Title | 1 | Identify the report as a systematic review. |  |
| ABSTRACT | | |  |
| Abstract | 2 | See the PRISMA 2020 for Abstracts checklist. |  |
| INTRODUCTION | | |  |
| Rationale | 3 | Describe the rationale for the review in the context of existing knowledge. |  |
| Objectives | 4 | Provide an explicit statement of the objective(s) or question(s) the review addresses. |  |
| METHODS | | |  |
| Eligibility criteria | 5 | Specify the inclusion and exclusion criteria for the review and how studies were grouped for the syntheses. |  |
| Information sources | 6 | Specify all databases, registers, websites, organisations, reference lists and other sources searched or consulted to identify studies. Specify the date when each source was last searched or consulted. |  |
| Search strategy | 7 | Present the full search strategies for all databases, registers and websites, including any filters and limits used. |  |
| Selection process | 8 | Specify the methods used to decide whether a study met the inclusion criteria of the review, including how many reviewers screened each record and each report retrieved, whether they worked independently, and if applicable, details of automation tools used in the process. |  |
| Data collection process | 9 | Specify the methods used to collect data from reports, including how many reviewers collected data from each report, whether they worked independently, any processes for obtaining or confirming data from study investigators, and if applicable, details of automation tools used in the process. |  |
| Data items | 10a | List and define all outcomes for which data were sought. Specify whether all results that were compatible with each outcome domain in each study were sought (e.g. for all measures, time points, analyses), and if not, the methods used to decide which results to collect. |  |
|  | 10b | List and define all other variables for which data were sought (e.g. participant and intervention characteristics, funding sources). Describe any assumptions made about any missing or unclear information. |  |
| Study risk of bias assessment | 11 | Specify the methods used to assess risk of bias in the included studies, including details of the tool(s) used, how many reviewers assessed each study and whether they worked independently, and if applicable, details of automation tools used in the process. |  |
| Effect measures | 12 | Specify for each outcome the effect measure(s) (e.g. risk ratio, mean difference) used in the synthesis or presentation of results. |  |
| Synthesis methods | 13a | Describe the processes used to decide which studies were eligible for each synthesis (e.g. tabulating the study intervention characteristics and comparing against the planned groups for each synthesis (item #5)). |  |
|  | 13b | Describe any methods required to prepare the data for presentation or synthesis, such as handling of missing summary statistics, or data conversions. |  |
|  | 13c | Describe any methods used to tabulate or visually display results of individual studies and syntheses. |  |
|  | 13d | Describe any methods used to synthesize results and provide a rationale for the choice(s). If meta-analysis was performed, describe the model(s), method(s) to identify the presence and extent of statistical heterogeneity, and software package(s) used. |  |
|  | 13e | Describe any methods used to explore possible causes of heterogeneity among study results (e.g. subgroup analysis, meta-regression). |  |
|  | 13f | Describe any sensitivity analyses conducted to assess robustness of the synthesized results. |  |
| Reporting bias assessment | 14 | Describe any methods used to assess risk of bias due to missing results in a synthesis (arising from reporting biases). |  |
| Certainty assessment | 15 | Describe any methods used to assess certainty (or confidence) in the body of evidence for an outcome. |  |
| RESULTS | | |  |
| Study selection | 16a | Describe the results of the search and selection process, from the number of records identified in the search to the number of studies included in the review, ideally using a flow diagram. |  |
|  | 16b | Cite studies that might appear to meet the inclusion criteria, but which were excluded, and explain why they were excluded. |  |
| Study characteristics | 17 | Cite each included study and present its characteristics. |  |
| Risk of bias in studies | 18 | Present assessments of risk of bias for each included study. |  |
| Results of individual studies | 19 | For all outcomes, present, for each study: (a) summary statistics for each group (where appropriate) and (b) an effect estimate and its precision (e.g. confidence/credible interval), ideally using structured tables or plots. |  |
| Results of syntheses | 20a | For each synthesis, briefly summarise the characteristics and risk of bias among contributing studies. |  |
|  | 20b | Present results of all statistical syntheses conducted. If meta-analysis was done, present for each the summary estimate and its precision (e.g. confidence/credible interval) and measures of statistical heterogeneity. If comparing groups, describe the direction of the effect. |  |
|  | 20c | Present results of all investigations of possible causes of heterogeneity among study results. |  |
|  | 20d | Present results of all sensitivity analyses conducted to assess the robustness of the synthesized results. |  |
| Reporting biases | 21 | Present assessments of risk of bias due to missing results (arising from reporting biases) for each synthesis assessed. |  |
| Certainty of evidence | 22 | Present assessments of certainty (or confidence) in the body of evidence for each outcome assessed. |  |
| DISCUSSION | | |  |
| Discussion | 23a | Provide a general interpretation of the results in the context of other evidence. |  |
|  | 23b | Discuss any limitations of the evidence included in the review. |  |
|  | 23c | Discuss any limitations of the review processes used. |  |
|  | 23d | Discuss implications of the results for practice, policy, and future research. |  |
| OTHER INFORMATION | | |  |
| Registration and protocol | 24a | Provide registration information for the review, including register name and registration number, or state that the review was not registered. |  |
|  | 24b | Indicate where the review protocol can be accessed, or state that a protocol was not prepared. |  |
|  | 24c | Describe and explain any amendments to information provided at registration or in the protocol. |  |
| Support | 25 | Describe sources of financial or non-financial support for the review, and the role of the funders or sponsors in the review. |  |
| Competing interests | 26 | Declare any competing interests of review authors. |  |
| Availability of data, code and other materials | 27 | Report which of the following are publicly available and where they can be found: template data collection forms; data extracted from included studies; data used for all analyses; analytic code; any other materials used in the review. |  |

*From:*  Page MJ, McKenzie JE, Bossuyt PM, Boutron I, Hoffmann TC, Mulrow CD, et al. The PRISMA 2020 statement: an updated guideline for reporting systematic reviews. BMJ 2021;372:n71. doi: 10.1136/bmj.n71

**Supplementary file 2: Literature search strategy**

**PubMed**

1. "Lupus Nephritis"[MeSH Terms] 8188
2. "lupus glomerulonephritis"[Title/Abstract] OR "nephritis lupus"[Title/Abstract] OR "lupus nephritides"[Title/Abstract] OR (("Nephritis"[MeSH Terms] OR "Nephritis"[All Fields] OR "Nephritides"[All Fields]) AND "Lupus"[Title/Abstract]) OR "glomerulonephritis lupus"[Title/Abstract] OR "glomerulonephritides lupus"[Title/Abstract] OR (("lupus vulgaris"[MeSH Terms] OR ("Lupus"[All Fields] AND "vulgaris"[All Fields]) OR "lupus vulgaris"[All Fields] OR "Lupus"[All Fields] OR "lupus erythematosus, systemic"[MeSH Terms] OR ("Lupus"[All Fields] AND "erythematosus"[All Fields] AND "systemic"[All Fields]) OR "systemic lupus erythematosus"[All Fields]) AND "Glomerulonephritides"[Title/Abstract]) 15159
3. 1 OR 2 15646
4. "drugs, chinese herbal"[MeSH Terms] 54051
5. ((("chineses"[All Fields] OR "east asian people"[MeSH Terms] OR ("east"[All Fields] AND "asian"[All Fields] AND "people"[All Fields]) OR "east asian people"[All Fields] OR "Chinese"[All Fields]) AND ("drug s"[All Fields] OR "pharmaceutical preparations"[MeSH Terms] OR ("pharmaceutical"[All Fields] AND "preparations"[All Fields]) OR "pharmaceutical preparations"[All Fields] OR "Drugs"[All Fields])) AND "Plant"[Title/Abstract]) OR "chinese herbal drugs"[Title/Abstract] OR (("herbal medicine"[MeSH Terms] OR ("Herbal"[All Fields] AND "medicine"[All Fields]) OR "herbal medicine"[All Fields] OR "herbalism"[All Fields] OR "Herbal"[All Fields] OR "herbals"[All Fields]) AND "drugs chinese"[Title/Abstract]) OR (("plant s"[All Fields] OR "planted"[All Fields] OR "planting"[All Fields] OR "plantings"[All Fields] OR "plants"[MeSH Terms] OR "plants"[All Fields] OR "Plant"[All Fields]) AND "extracts chinese"[Title/Abstract]) OR "chinese plant extracts"[Title/Abstract] OR (("extract"[All Fields] OR "extract s"[All Fields] OR "extractabilities"[All Fields] OR "extractability"[All Fields] OR "extractable"[All Fields] OR "extractables"[All Fields] OR "extractant"[All Fields] OR "extractants"[All Fields] OR "extracted"[All Fields] OR "extractibility"[All Fields] OR "extractible"[All Fields] OR "extracting"[All Fields] OR "extraction"[All Fields] OR "extractions"[All Fields] OR "extractive"[All Fields] OR "extractives"[All Fields] OR "Extracts"[All Fields]) AND "chinese plant"[Title/Abstract]) 2227
6. 4 OR 5 55217
7. "astragalus plant"[Title/Abstract] OR "Astragalus"[Title/Abstract] 4034
8. 6 OR 7 58450
9. 3 AND 8 51

**Embase**

#1. 'Lupus Nephritis'/exp 22177

#2. 'Lupus Nephritis':ab,ti 17564

#3. 'lupus glomerulonephritis':ab,ti 6421

#4. 'nephritis lupus':ab,ti 24226

#5. 'lupus nephritides':ab,ti 32

#6. 'glomerulonephritis lupus':ab,ti 8099

#7. 'glomerulonephritides lupus':ab,ti 107

#8. 1 OR 2 OR 3 OR 4 OR 5 OR 6 OR7 31626

#9. 'drugs, chinese herbal':ab,ti 4095

#10. 'Chinese Herbal Drugs':ab,ti 4910

#11. 'Astragalus':ab,ti 4761

#12. 'Astragalus Plant':ab,ti 834

#13. 9 OR 10 OR 11 OR 12 10617

#14. 8 AND 13 13

**Web of science**

1. TS=(Lupus Nephritis) 32491
2. TS=(lupus glomerulonephritis) 17434
3. TS=(nephritis lupus) 32491
4. TS=(lupus nephritides) 68
5. TS=(glomerulonephritis lupus) 17434
6. TS=(glomerulonephritides lupus) 17434
7. #1 OR #2 OR #3 OR #4 OR #5 OR #6 41531
8. TS=(Chinese Herbal Drugs) 84556
9. TS=(drugs, chinese herbal) 80542
10. TS=(Astragalus) 78084
11. TS=(Astragalus Plant) 14666
12. #8 OR #9 OR #10 OR #11 154960
13. #7 AND #12 113

知网：

（主题：狼疮性肾炎 + '狼疮性肾炎(ln)' + 狼疮性肾炎疾病 + 狼疮性肾炎肾病综合征 + 狼疮性肾炎病） AND (主题：黄芪 + 黄芪药材 + '黄芪多糖(aps)' + 复方黄芪 + 中药材黄芪 + 黄芪注射液) 351

万方：

[主题:(狼疮性肾炎 or 狼疮性肾炎疾病 or 狼疮性肾炎肾病综合征 or 狼疮性肾炎病) and 主题:(黄芪 or 黄芪药材 or 黄芪多糖 or 复方黄芪 or 中药材黄芪 or 黄芪注射液)](https://s.wanfangdata.com.cn/advanced-search/paper?q=%E4%B8%BB%E9%A2%98:(%E7%8B%BC%E7%96%AE%E6%80%A7%E8%82%BE%E7%82%8E%20or%20%20%E7%8B%BC%E7%96%AE%E6%80%A7%E8%82%BE%E7%82%8E%E7%96%BE%E7%97%85%20or%20%E7%8B%BC%E7%96%AE%E6%80%A7%E8%82%BE%E7%82%8E%E8%82%BE%E7%97%85%E7%BB%BC%E5%90%88%E5%BE%81%20or%20%E7%8B%BC%E7%96%AE%E6%80%A7%E8%82%BE%E7%82%8E%E7%97%85)%20and%20%E4%B8%BB%E9%A2%98:(%E9%BB%84%E8%8A%AA%20or%20%E9%BB%84%E8%8A%AA%E8%8D%AF%E6%9D%90%20or%20%E9%BB%84%E8%8A%AA%E5%A4%9A%E7%B3%96%20or%20%E5%A4%8D%E6%96%B9%E9%BB%84%E8%8A%AA%20or%20%E4%B8%AD%E8%8D%AF%E6%9D%90%E9%BB%84%E8%8A%AA%20or%20%E9%BB%84%E8%8A%AA%E6%B3%A8%E5%B0%84%E6%B6%B2)&searchtype=expert&type=%5b) 218

**Supplementary file 3:** The detailed information of herbal medicines in intervention group in each RCT.

| **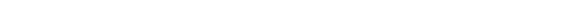Study** | **Constituent drugs in Astragalus -containing herbal formulas** | **Dosage and number of doses administered daily** | **Route of administration** |
| --- | --- | --- | --- |
| Guishiyuan 2022 | Astragalus 30g, Codonopsis pilosula 20g, Millettia reticulata 30g, Leonurus japonicus 20g, Rehmannia glutinosa 15g, Chinese yam 20g, Cornus officinalis 15g, Poria 20g, Paeonia suffruticosa 15g, Orientia 15g, Hedyotis diffusa 15g, Pheretima lum 20g, Licorice root 6g | One dose per day, one dose is decocted into two bags, one bag has a dosage of 200ml, take two bags per day | Oral decoction |
| Zhu Aimin 2010 | Astragalus Injection (Chengdu Di Ao Jiuhong Pharmaceutical Factory, National Drug Z51021776) | 50 mL, Once a day | Intravenous injection |
| Qu Huanru 2010 | Chinese Herbal Compound Formula for Tonifying Kidney and Consolidating Essence (Astragalus, Radix Rehmanniae Praeparata, Curcuma longa, Radix Polygoni Multiflori, Cicada Flower, Cuttlebone) and Astragalus Injection | 40 mL, Once a day | Intravenous injection |
| Xie Chao 2010 | Yishen Huoxue Decoction (Astragalus, Radix et Rhizoma Ginseng, Radix Rehmanniae Praeparata, Radix Achyranthis Bidentatae, Radix et Rhizoma Rhei) | 150 mL, Once a day | Oral decoction |
| Li Gui’an 2006 | Raw Astragalus 30-60g, Radix Rehmanniae Praeparata, Radix Angelicae Sinensis, Radix Paeoniae Alba, Radix Rehmanniae Praeparata, Salviae Miltiorrhizae 15g, Rhizoma Ligustici Chuanxiong 10g | Not reported | Oral decoction |
| Zhang Minghua 2008 | Astragalus Injection 30ml + Danshen Injection 10ml | 30ml, 10ml | Intravenous injection |
| Geyang 2022 | Astragalus, Poria, Atractylodes Macrocephala, Radix Rehmanniae Praeparata, Semen Cuscutae Chinensis, Semen Cherry, Fructus Raspberry | 400 mL, Twice daily | Oral decoction |
| Xiang Caichun 2008 | Astragalus 20g, Rehmannia glutinosa 15g, Chinese yam 15g, Cornus officinalis 10g, Oldenlandia diffusa 15g, Salvia miltiorrhiza 15g, Scutellaria barbata 10g, Chrysanthemum 10g, Paeonia suffruticosa 15g, Carthamus tinctorius 5g, Ligustrum lucidum 15g, Lycium barbarum 15g, Panax notoginseng 5g | 1 dose per day, 500 mL | Oral decoction |
| Lu Sihao 2019 | Raw Astragalus 30g, Radix Rehmanniae Praeparata 15g, Fructus Schisandrae Chinensis 15g, Artemisiae argyi 15g, Rhizoma Polygonati Odorati 15g, Salviae Miltiorrhizae 10g, Rhizoma Ligustici Chuanxiong 10g, Fructus Schisandrae chinensis 10g, Radix Paeoniae Lactiflorae 20g, Radix et Rhizoma Rhei 10g, Rhizoma Rhei Charcoal 10g, Rhizoma Puerariae Charcoal 10g, Rhizoma Atractylodes Macrocephalae 15g | 1 dose per day | Oral decoction |
| Yang Yeying 2019 | Astragalus 30 g, Radix et rhizoma 15 g, Radix chasteberry 30 g, Radix aristolochiae 30 g, Radix Panax quinquefolium 15 g, Curcuma longa 15 g, Radix et rhizoma alba 30 g, Radix chrysanthemum officinale 15 g | 1 dose per day, 400 mL | Oral decoction |
| Chen Xiangjun 2003 | Astragalus Membranaceus, Cuttlebone, Radix Rehmanniae Praeparata, Cortex Eucommiae, Salviae Miltiorrhizae, Curcuma longa, Fructus Jinchuanzi | Not reported | Oral decoction |
| Su Li 2007 | Astragalus Injection, manufactured by Shanghai Fuda Pharmaceutical Factory (10 ml/bolus, Lot 970913) | 10 ml/bolus | Intravenous injection |
| Zhong Li 2007 | Radix Rehmanniae Praeparata 20 g, Cornu Cervi Pantotrichum 15 g, Rhizoma Dioscoreae 10 g, Rhizoma Zedoariae 10 g, Radix Paeoniae Miltiorrhizae 10 g, Poria Cocos 30 g, Fructus Lycium Chinense 15 g, Radix Astragali 30 g, Radix Aesculus Spleen 10 g, Rhizoma Pseudostemonis Macrocephala 10 g | 100 ml | Oral decoction |
| Yuan Xiaoying 2021 | Astragalus (Zhengda Youthful Treasure Pharmaceutical Co.Z33020179) | 10 ml | Intravenous injection |


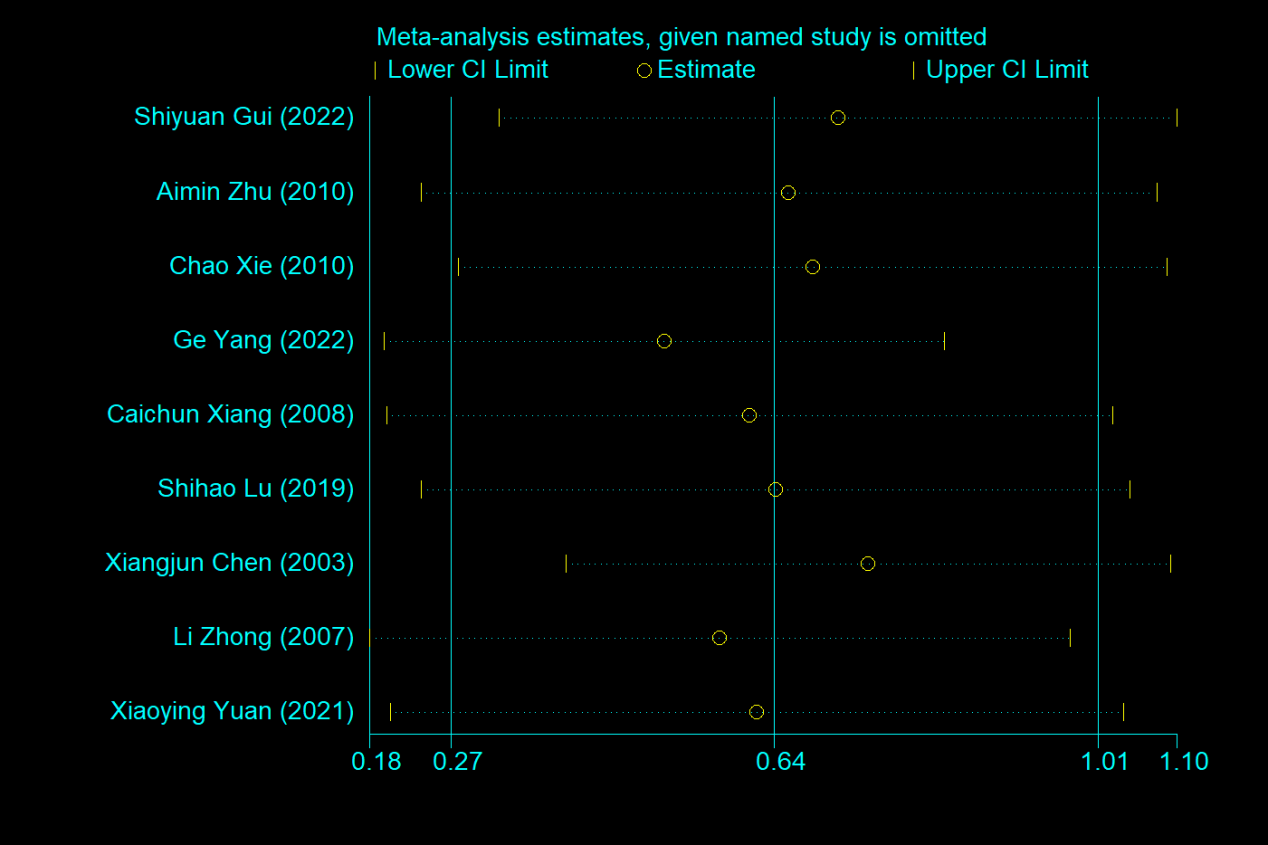


**Supplementary file 4:** The sensitivity analysis for SCr levels.


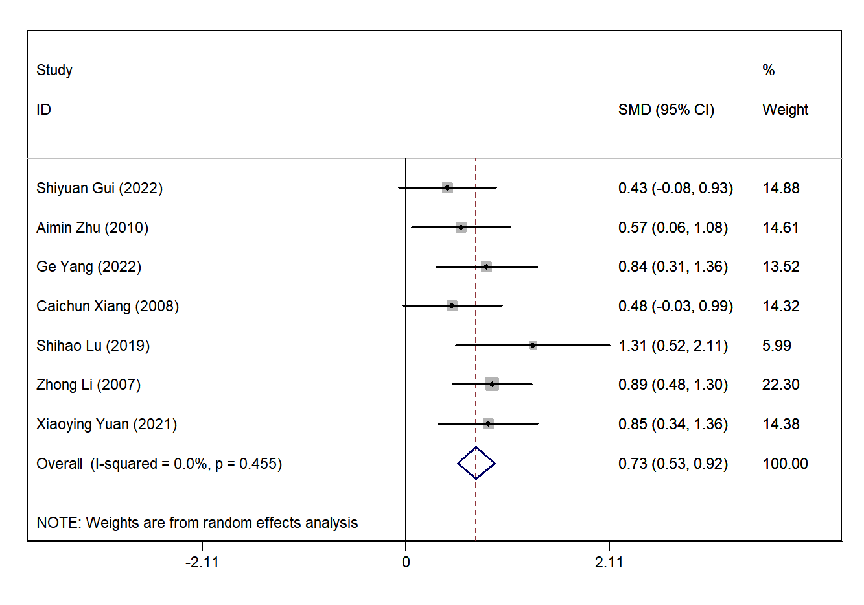


**Supplementary file 5:** The forest plot showing the effect of CHM containing Astragalus with Western treatment on BUN.


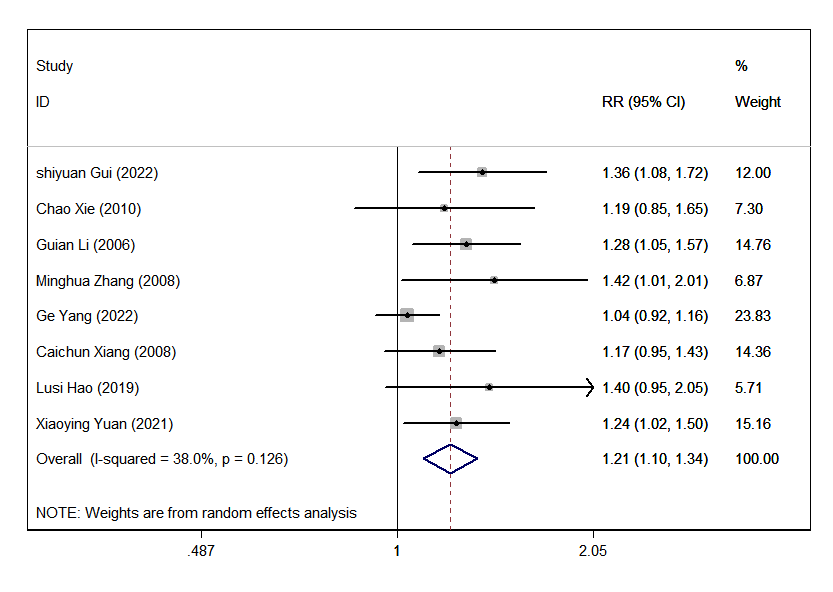


**Supplementary file 6:** The forest plot showing the effect of CHM containing Astragalus with Western treatment on ORR.


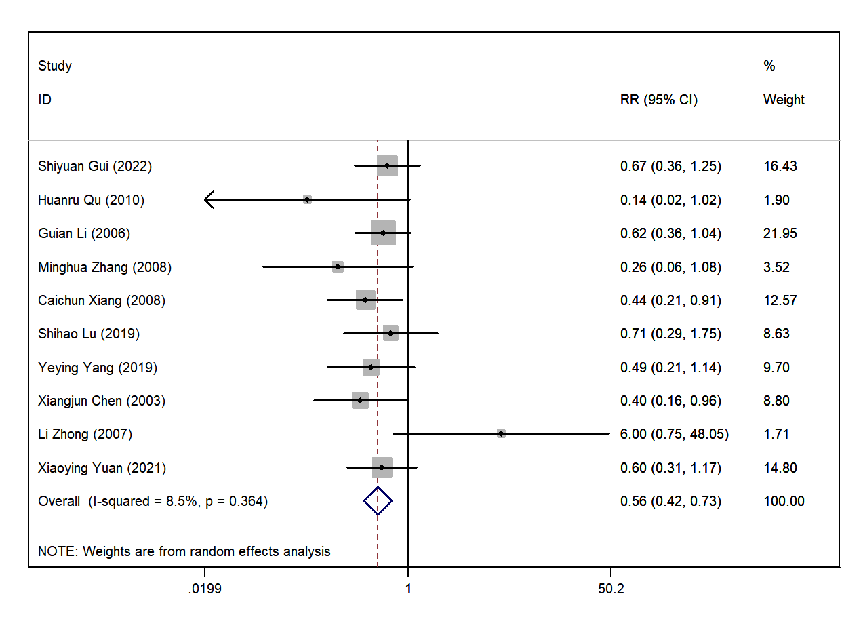


**Supplementary file 7:** The forest plot showing the effect of CHM containing Astragalus with Western treatment on adverse events.


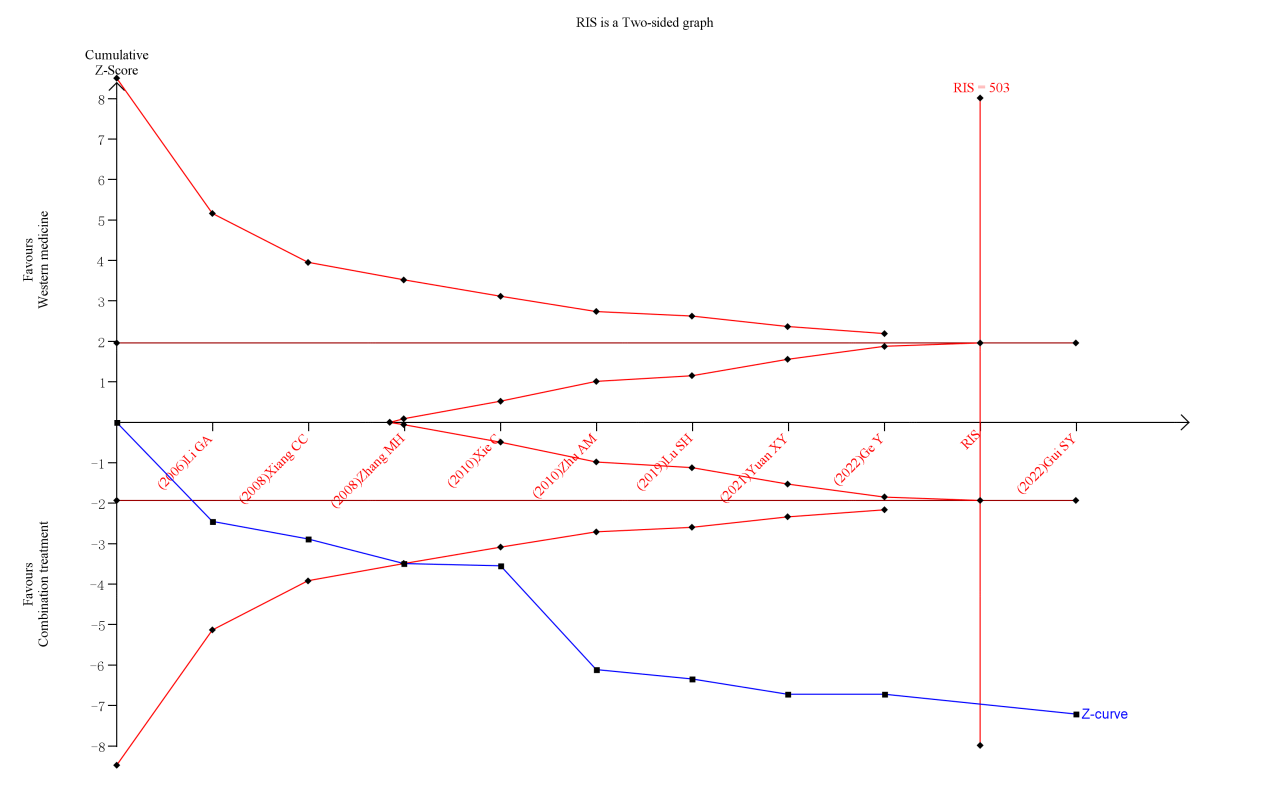


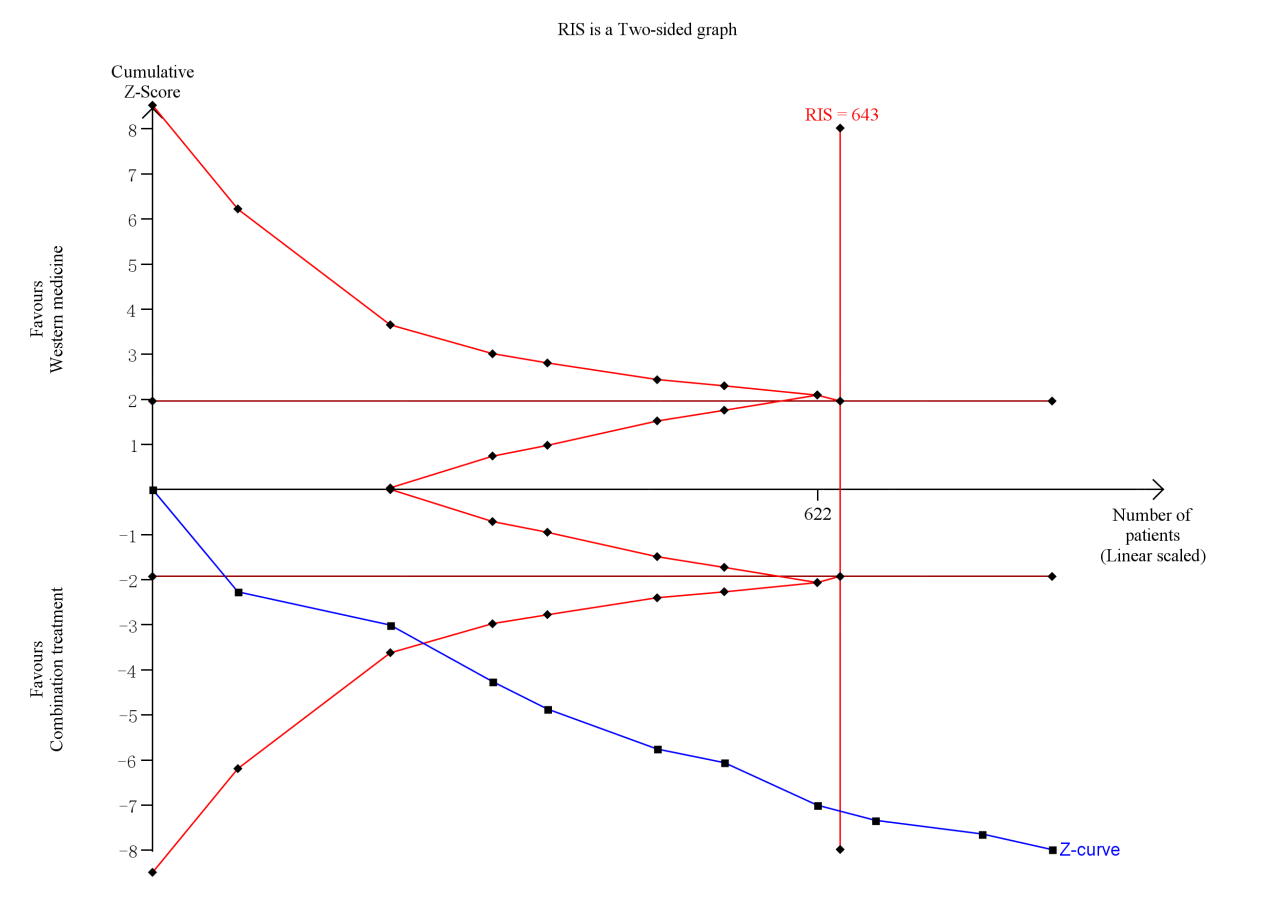


**Supplementary file 8:** Trial sequential analysis of fourteen trials for objective ORR and AEs. The cumulative Z curve crossed both the conventional boundary and the trial sequential monitoring boundary, and reached the required information size. A: for ORR, the required information size of 503 patients was calculated by using an event proportion of 26.60% in the control group. B: for AEs, the required information size of 643 patients was calculated by using an event proportion of 55.25% in the control group.
